# Protocol for The Promoting Resilience in Stress Management (PRISM) Intervention: a multi-site randomized controlled trial for adolescents and young adults with advanced cancer

**DOI:** 10.21203/rs.3.rs-2748874/v1

**Published:** 2023-04-07

**Authors:** Alison O’Daffer, Liam Comiskey, Samantha R. Scott, Chuan Zhou, Miranda C. Bradford, Joyce P. Yi-Frazier, Abby R. Rosenberg

**Affiliations:** Seattle Children’s Research Institute; Seattle Children’s Research Institute; University of Denver; University of Washington; Seattle Children’s Research Institute; Seattle Children’s Research Institute; Dana-Farber Cancer Institute

**Keywords:** Cancer, Quality of Life, Anxiety, Depression, Hope, Coping Skills, Communication

## Abstract

**Background:**

Adolescents and young adults (AYAs) with cancer are at high risk of poor psychosocial outcomes, and evidence-based interventions designed to meet their psychosocial and communication needs are lacking. The main objective of this project is to test the efficacy of a new adaptation of the Promoting Resilience in Stress Management intervention for AYAs with Advanced Cancer (PRISM-AC).

**Methods/design:**

The PRISM-AC trial is a 2-arm, parallel, non-blinded, multisite, randomized controlled trial. 144 participants with advanced cancer will be enrolled and randomized to either usual, non-directive, supportive care without PRISM-AC (“control” arm) or with PRISM-AC (“experimental” arm). PRISM is a manualized, skills-based training program comprised of four 30–60 minute, one-on-one sessions targeting AYA-endorsed resilience resources (stress-management, goal-setting, cognitive-reframing, and meaning-making). It also includes a facilitated family meeting and a fully equipped smartphone app. The current adaptation includes an embedded advance care planning module. English- or Spanish-speaking individuals 12–24 years old with advanced cancer (defined as progressive, recurrent, or refractory disease, or any diagnosis associated with < 50% survival) receiving care at 4 academic medical centers are eligible. Patients’ caregivers are also eligible to participate in this study if they are able to speak and read English or Spanish, and are cognitively and physically able to participate. Participants in all groups complete surveys querying patient-reported outcomes at the time of enrollment and 3-, 6-, 9-, and 12-months post-enrollment. The primary outcome of interest is patient-reported health-related quality of life (HRQOL) and secondary outcomes of interest include patient anxiety, depression, resilience, hope and symptom burden, parent/caregiver anxiety, depression and health-related quality of life, and family palliative care activation. We will conduct intention-to-treat analysis to compare the group means of primary and secondary outcomes between PRISM-AC arm and control arm with regression models.

**Discussion:**

This study will provide methodologically rigorous data and evidence regarding a novel intervention to promote resilience and reduce distress among AYAs with advanced cancer. This research has the potential to offer a practical, skills-based curriculum designed to improve outcomes for this high-risk group.

## Introduction

Adolescents and Young Adults (AYAs) with cancer are at high risk of poor psychosocial outcomes, as cancer disrupts normal developmental experiences like establishment and identification of personal, social, and sexual identity, and pursuit of educational and vocational goals.^1–5^ Unmet needs (e.g., inadequate psychosocial support and lack of information about disease management) can further contribute to poor outcomes, including poor health-related quality of life (HRQOL).^6–9^

Among patients with cancer and their families, early integration of palliative care can improve quality of life. This is particularly important for AYAs because their distinct developmental challenges related to identity, relationships, and vocation may add to the burden of cancer.^1–5^ Among AYAs with advanced cancer, most understand that they may die and report that discussing end-of-life preferences, goals, and fears would be helpful; however, only 53% engage in such conversations.^10–12^ While national guidelines call for integrated palliative care in AYA oncology,^13–15^ developmentally targeted, evidence-based interventions designed to meet psychosocial and communication needs are lacking.

A potential barrier to improving the experiences of AYAs with advanced cancer is their limited opportunities to develop “resilience resources” such as stress-management, goal-setting, positive reframing, and meaning-making skills.^16^ These resources can mitigate negative outcomes, facilitate engagement in goals of care discussions, and improve quality of life.^17–19^ Furthermore, promoting these resources among AYAs can give them the tools to more successfully navigate the challenges of the cancer experience.

The present proposal is built on increasing evidence that promoting resilience resources will improve psychosocial well-being. Over a series of studies, we developed a conceptual framework of resilience in pediatric cancer,^16,20^ affirmed associations between resilience resources and outcomes,^21^ and developed a novel resilience resources intervention (Promoting Resilience in Stress Management, PRISM).^22^ PRISM is a manualized, skills-based training program comprised of four 30–60 minute, one-on-one sessions plus a facilitated parent/caregiver/spouse/significant other family meeting. We developed PRISM based on stress and coping theory to be a brief, skills-based intervention targeting AYA resilience resources. Results from a phase II randomized controlled trial (RCT) demonstrate it is associated with increased AYA patient-reported resilience and HRQOL. It also provides opportunities for AYAs to articulate goals and meaning from their cancer experience, thereby facilitating communication and patient-activation. Qualitative feedback from patients with advanced cancer suggested refinements targeting hopes, worries, and contextual meaning-making might strengthen PRISM’s usefulness.

Based on this feedback, we developed “PRISM-AC” for patients with advanced cancer. The adapted program includes an additional module focused on advance care planning. Specifically, it incorporates the age-validated *Voicing my Choices* (VMC) advance care planning guide and provides opportunities to explore and communicate patient-endorsed values and priorities to loved ones and care-teams. We conducted a pilot trial at a single site testing the feasibility and acceptability of PRISM-AC.^23^ Results showed that, of 26 enrolled and randomized AYAs with advanced cancer, 82% completed the entire PRISM intervention including VMC, surpassing an a priori threshold for feasibility of ≥70% completion. Feedback was highly positive, with 100% of participants describing the entire program as valuable, and 91% describing the VMC session, specifically, as valuable. This pilot trial demonstrated that integrating advanced care planning into resilience coaching was both feasible and acceptable. Taken together, the background literature and our prior experiences underscored a critical knowledge gap: How might PRISM-AC support the wellbeing of AYAs with advanced cancer? To answer this question, we designed a multi-site randomized controlled trial. Here, we describe the protocol of that ongoing study.

## Methods/design

The primary aim of this study is to evaluate the efficacy of PRISM-AC compared to usual care (UC) on AYA-reported Health-Related Quality of Life (HRQOL, measured by the Pediatric Quality of Life (PedsQL) Generic and Cancer Module Teen Reports scales^24,25^) 3-months post-enrollment. We hypothesize that PRISM-AC will be associated with higher HRQOL compared to UC. We additionally aim to evaluate the impact of PRISM-AC on other key patient-reported outcomes 3-months following enrollment, including symptom burden (measured by the Memorial Symptom Assessment Scale (MSAS)^26,27^), anxiety and depression (measured by the Hospital Anxiety and Depression Scale (HADS)^28^), hope (measured by the Snyder “Hope” Scale^29^), and resilience (measured by the Connor-Davidson Resilience Scale (CD-RISC 10)^30,31^). We hypothesize that PRISM-AC will be associated with lower total symptom burden; lower anxiety; lower depression; higher hope; and higher resilience, compared to UC.

Additional secondary and exploratory aims include: (a) to evaluate PRISM-AC’s impact on parent/caregiver health-related quality of life (measured by the Medical Outcomes Study Rand 36-item Health Survey (SF-36)^32^), anxiety (measured by the Generalized Anxiety Disorder Screener (GAD-7)^33,34^) and depression symptoms (measured by the Patient Health Questionnaire 8 (PHQ-8)^35–40^) 3-months following enrollment; (b) to evaluate the impact of PRISM-AC on family “palliative care activation” (measured by the Decision Making Involvement Scale (DMIS)^41^ and the Survey of Caring for Children with Cancer (SCCC)^42–44^); and (c) to evaluate the longitudinal impact of PRISM-AC on all AYA/parent-reported outcomes at 6-, 9-, and 12-months following enrollment.

### Trial Design

**Figure 1:**
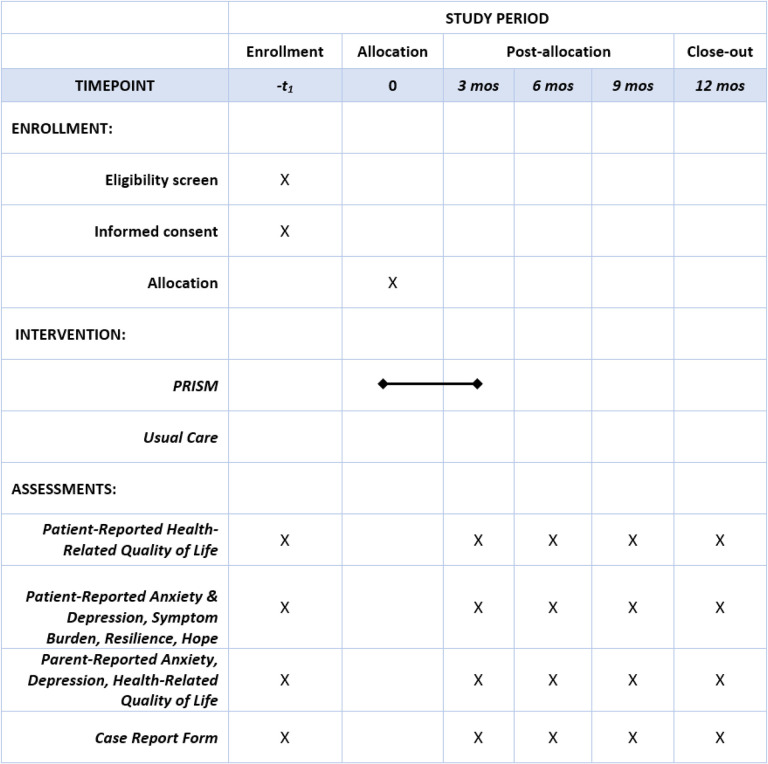
Study Activities.

The PRISM-AC trial is a parallel, 2-arm, non-blinded multisite randomized controlled trial. All study activities are outlined in Fig. 1. 144 participants with advanced cancer will be enrolled and randomized to either usual, non-directive, supportive care without PRISM-AC (“control” arm) or with PRISM-AC (“experimental” arm). Randomization will be stratified by age (patients ages 12–17 versus ages 18–24) and site. Patients will be randomized only after completion of baseline surveys and in a 1:1 ratio to control arm and experimental arm. Biostatisticians who will conduct data analysis will be blinded from the treatment group allocations. Due to the nature of the PRISM-AC intervention, patients cannot be blinded to study arm assignment.

### Participants

Individuals are eligible if they are 12–24 years old, are able to speak English and read English or Spanish, are receiving cancer care at Seattle Children’s Hospital, Texas Children’s Hospital/Baylor College of Medicine, Children’s Hospital Los Angeles, or University of Pittsburgh Medical Center Children’s Hospital, are cognitively able to participate in PRISM sessions and surveys, and have been diagnosed with advanced cancer (defined as progressive, recurrent, or refractory disease, or any diagnosis associated with < 50% survival) or a progressive desmoid tumor at least 2 weeks prior to enrollment.

Parents, caregivers, or guardians of AYA patient participants will be eligible to complete surveys as part of this study. To participate, they must be able to speak and read English or Spanish and be cognitively and physically able to participate. Only one parent/caregiver or guardian per AYA can complete surveys. Concurrent parent/caregiver participation is not required for AYA patient participation, and parent survey completion is not required for AYA patient participation.

### Sample Size

The target analytic sample size is N = 144 AYA participants (72/arm). Based on our prior work and characteristics of AYAs at participating centers, we estimate identifying a total of 120 eligible AYAs within a 12-month window (300 over the 30-months of enrollment). With a conservative attrition rate of 26% (due to medical complications or death as seen in pilot), we plan to enroll N = 200 participants and hope to have complete data collection on 144 AYAs (72/arm). This sample size achieves 80% power to detect an increase of 8.1 in mean total PedsQL score, the main study outcome.

Parents/caregivers will complete surveys at the same time points as the AYAs. In our prior studies including AYA-parent dyads, > 90% of caregivers participated. Hence, we expect complete data from a minimum of 128 caregivers (64/arm). Only one caregiver will be invited to complete questionnaires, and will be designated at enrollment.

### Recruitment

We will recruit AYAs and their primary caregivers from outpatient clinics and inpatient wards of 4 pediatric academic medical institutions across the country. Research Associates (RAs) at each site will screen patients via review of medical records and verify the patient’s diagnosis with a trained oncology provider. Recruitment will occur either in person (inpatient hospital rooms and/or outpatient clinic) or by phone/video call (i.e., direct contact by study staff). Patients will either be approached inpatient, in clinic, or remotely.

### The Promoting Resilience in Stress Management (PRISM) Intervention

Participants randomized to the intervention arm receive the Promoting Resilience in Stress Management (PRISM) intervention. PRISM consists of four, 30–50 minute, one-on-one sessions approximately 1–2 weeks apart, plus a session for AYA and caregivers, together (Table 1). Supplemental materials (e.g., media-links to resources, worksheets, text-based reminders, and a digital app to track and practice skills) are provided between sessions. The digital app is an interactive platform to practice the same PRISM exercises that are taught in the PRISM intervention script.

To increase translation and wider application of PRISM in the future, a trained non-clinical research associate administers it, as described in previous models and our pilot studies.^22,45^ The 1st session occurs within 2 weeks of enrollment, and other sessions are scheduled around patient clinic and/or hospital visits (depending on concurrent illness and medical needs). Following the “Coming Together” session, intervention participants will be offered every other week “booster” contacts until they reach the 3 month point from enrollment. Sessions are done in-person (in clinic or inpatient), via phone or via other web-based communication (i.e. Zoom, WebEx).

Details of the sessions are listed in Table 1. Sessions 1–4 cover the topics of stress-management, goal-setting, cognitive restructuring, and benefit finding. For this study and the development of PRISM-AC, we add a 5th main session focused on early advance care planning, using the age-validated and widely used Voicing My Choices: A Planning Guide for Adolescents and Young Adults (VMC^46^. Briefiy, VMC was designed in partnership with AYA patients, parents, and Aging with Dignity (agingwithdignity.org) to mimic legal advance directives available for older adults. The document has been tested for feasibility and acceptability among AYAs with cancer and other life-limiting illness and is the established standard of care for advance care planning in this age group. The instrument is designed to be introduced by trained staff (including research staff and non-medical personnel), and completed by AYAs either with such staff present, with family, or independently, depending on AYA preferences.

Because PRISM sessions 1–4 build skills to identify goals and values, VMC is an appropriate culmination of skills and offers concrete examples of how to utilize them. For this reason, PRISM-AC introduces specific VMC pages as examples of how such skills might be helpful in cases of advanced cancer, and then offer opportunities to practice them in real time. Coaches will only complete up to four specific pages with AYAs: page 4 “My Comfort”, page 5 “My Support”, page 8 “My Friends and Family to Know”, and page 9 “My Spiritual Thoughts”. If AYAs express a desire to do any of the other pages, we will redirect them to their medical team, social work, and/or their parents. Specifically, we will introduce the VMC booklet and then highlight the pages and allow participants to choose the one(s) that resonate with them “a la carte.” Although advance care planning includes sensitive topics like end-of-life planning, this booklet was selected specifically because they are more generic and applicable to all AYAs with illness. If an AYA has no specific preferences, the coach will direct the patient to the page about “my comfort” (VMC page 4) and complete that page during the session. Staff will ask what they would like to share with their parent(s), spouses, significant others, or guardians during the final session such that the coaches can prepare to facilitate the final session.

The final session (“Coming Together”) allows patients to reflect on the skills they have learned, to identify those that resonate and work for them, and to share their thoughts with parents, family-members, and loved ones. Patients may opt out of the “Coming Together” session if they request to do so explicitly and do a booster session instead.

PRISM sessions are audio-recorded and scored for fidelity using a standardized tool by a supervising licensed clinical psychologist. Coaches receive biweekly 1:1 supervision, which includes feedback and, if necessary, re-training to address fidelity concerns.

### Procedure

#### Baseline Survey Completion

Upon enrollment, study staff will deliver the baseline survey in participant’s preferred language (English or Spanish). The survey will first be offered by email via the Research Electronic Data Capture web-based application^47,48^ (REDCap) or via REDCap on a study team iPad. Upon request, staff will offer paper-pencil versions and/or interview-based versions.

### Measures

#### Patient-Reported Outcome Surveys

At enrollment, 3-months, 6-months, 9-months, and 12-months, AYAs and parents/caregivers on both arms will complete a comprehensive survey of age-appropriate validated instruments and standard demographics. Baseline surveys must be completed within two weeks of enrollment. Subsequent surveys must be completed within 28 days of their due date. Participants are given weekly reminders via phone, email, or in-person until surveys are completed. AYA participants are paid a total of $50 for survey completion.

Our primary outcome is patient-reported health-related quality of life (HRQOL) measured using the Pediatric Quality of Life (PedsQL) Generic and Cancer Module Teen Reports scale. The PedsQL 4.0 Generic and 3.0 Cancer Module include 50 items evaluating HRQOL of AYAs with cancer. Queries assess physical, emotional, social, and school well-being, plus cancer-related pain and hurt, nausea, procedural anxiety, treatment anxiety, worry, cognitive problems, perceived physical appearance, and communication. Scales are available for teens and young adults,^24,25^ and internal consistency ranges from 0.75 to 0.92.^24^ Items are rated on a 5-point Likert scale and total scores transformed to a 0–100 scale with higher scores representing better HRQOL.

Secondary outcomes for AYAs include (a) Anxiety and depression, as measured by the Hospital Anxiety and Depression Scale (HADS).^28^ The HADS assesses mixed affective symptoms in patients with serious illness.^28^ (b) Symptom burden, as measured by the Memorial Symptom Assessment Scale (MSAS).^26,27^ This instrument assesses the presence, severity, frequency, and extent of bother from 26 symptoms.^49,50^ (c) Hope, as measured by the Snyder “Hope” Scale. This instrument contains 8 hope items plus 4 “filler” questions and measures “the overall perception that one’s goals can be met.”^29^ (d) Resilience, as measured by the Connor-Davidson Resilience Scale (CD-RISC 10), a reliable and widely used instrument to measure inherent resiliency.^30,31^

Parent outcomes include (a) anxiety, as measured by the Generalized Anxiety Disorder Screener (GAD-7). The GAD-7 is a 7-item survey commonly used to identify cases of generalized anxiety disorder and to assess symptom severity.^33,34^ (b) Depression, as measured by the Patient Health Questionnaire 8 (PHQ-8). The PHQ-8 is an 8-item survey is widely used among general populations, patients with chronic illness, and in parents of children with cancer to assess degree of depression.^35–40^ (c) Health-related quality of life (HRQOL), as measured by the Medical Outcomes Study Rand 36-item Health Survey (SF-36). The SF-36 incorporates 8 concepts: physical functioning, body pain, limitations due to physical health problems, role limitations due to personal or emotional problems as well as emotional well-being and social functioning, energy, fatigue and general health perceptions to evaluate HRQOL.^32^

#### Medical Record Abstraction

Trained study staff at each site will abstract information from the medical record using a study-specific case report form (CRF). Variables abstracted will include: (1) AYA participation in goals of care conversations: dates of documented conversations with medical team regarding prognosis, treatment decisions, and/or goals of care, whether or not AYA was present, and if there is documented active AYA participation. (2) Benchmarks of Palliative Care Utilization: number and frequency of documented psychosocial and palliative care referrals and meetings, hospice referrals, limitation-of-resuscitation orders, completion of advance care planning documents, and end of life details (i.e. location of death, clinical involvement and family support (sibling & financial assessments)). (3) Clinical covariates: the AYA’s diagnosis, cancer/tumor-directed treatments, and intensity in the past month,^27^ number of and reason for hospital days (anticipated and unanticipated), prescription psychiatric and/or mood-altering medications, prescription opioids and other pain medications, and number of documented palliative care/psychosocial encounters.

### Data Analysis

#### Primary Outcomes Analyses

Our primary outcome is AYA-reported HRQOL at 3-months. Because the amount of change depends strongly on the initial HRQOL at baseline we will control for baseline HRQOL as a covariate in the regression. Linear regression models will be used to estimate adjusted mean differences in 3-month outcomes between the PRISM-AC arm and usual care arm, and associated 95% confidence intervals. In the regression model, for example, the total PedsQL generic core score will be the outcome, the PRISM intervention will be the predictor of interest, and baseline PedsQL and study site as covariates. Wald t-test will be used to assess if there is an increase in PedsQL scores from baseline for PRISM versus usual care at the primary time point of interest, 3-months following enrollment. The same analysis will be undertaken for the domain subscales of PedsQL (physical and psychosocial), the cancer-specific module, and secondary outcomes included in AYA surveys. Outcomes assessed longitudinally will be analyzed using linear mixed effects models with subject specific random effect to account within-subject correlations. We will estimate the fixed effects using restricted maximum likelihood (REML) method and test their significance with Kenward-Roger’s approximation of the degrees of freedom.^51^ Subgroup analyses will explore whether the effect of the intervention is modified by medical covariates, symptom distress, and/or concurrent parent distress. Response type (survey, interview, or parent proxy) will be included in sensitivity analyses and data reported separately if indicated. The rationale for these subgroup analyses is grounded in prior findings suggesting symptoms and parent wellbeing are associated with patient HRQOL.^52,53^

While our goal is to minimize missing data, data may still be missing due to participants skipping individual survey items, omissions in medical records, lack of follow-up, medical complications, or death. We will quantify the amount of missing data, evaluate the pattern of missingness, association of participant characteristics with missing data, and minimize bias and increase efficiency in the associations of interest by applying appropriate methods to account for missing data.^54–56^ For example, for outcomes where missing at random (MAR) is a plausible assumption, we will use multiple imputation techniques and conduct regression models on the imputed datasets then report pooled results. For missing not at random (MNAR) data, we will perform additional sensitivity analyses. In all cases, we will assess the robustness of estimates due to assumptions.

#### Data Safety and Monitoring

Data safety and monitoring is conducted by a 4-member Data Safety and Monitoring Committee (DSMC) independent of the protocol. The committee is convened twice annually to provide input and guidance on the study evaluation and intervention protocols and data handling activities. DSMC members provide input and feedback to the PI and co-investigators related to (a) accrual rate, (b) study eligibility determination issues, (c) data completion rates including conformance with informed consent requirements, (d) intervention fidelity indicators, (e) adverse events, and (f) compliance with data management procedures. This study does not have pre-set stopping rules, but the DSMC has the option of requesting the data be un-blinded and may alter the study or stop the study early.

## Discussion

This RCT is designed to assess the efficacy of the PRISM Advanced Cancer intervention, a brief skills-based intervention based on stress and coping theory targeting AYA resilience resources. This specialized version of the program also provides opportunities for AYAs to articulate goals and meaning from their cancer experience, thereby facilitating communication and patient-activation. This intervention was iteratively designed based on input from AYAs with chronic illnesses, and the design of this trial is in direct response to advanced cancer patients’ requests for more diagnosis-specific content. We hope that the inclusion of advanced care planning in PRISM-AC will bolster the program’s effectiveness for this population and their caregivers.

### Strengths & Limitations

This study has several important strengths. First, few evidence-based psychosocial supportive care programs exist to provide both psychosocial supportive care and communication skills for AYAs with advanced cancer. The integration of advanced care planning into the previously successful PRISM program addresses a specific gap for AYAs with advanced cancer. Second, this trial includes four sites, adding more geographic and demographic diversity than prior PRISM studies.

There are also several pertinent limitations of this trial protocol. First, this study aims to learn about AYAs with advanced cancer, a population seriously impacted by illness severity. Patients and families’ stress associated with treatment and illness may impact their willingness to enroll in the study. We expect significant attrition due to critical illness or death, which may affect session and survey completion. Second, the control condition for this study does not involve a sham intervention,, limiting our ability to evaluate what part of PRISM’s efficacy is related to 1:1 relational contact with a PRISM coach. Third, while surveys are available in English or Spanish for both AYAs and parents, AYAs must be proficient in spoken English to participate in the PRISM program. Results may be less generalizable due to limited inclusion criteria related to language proficiency. Future studies should translate, adapt, and validate PRISM into other languages.

## Conclusion

Given high levels of distress among AYAs with advanced cancer and their parents/caregivers, supportive care interventions to address these needs are necessary. If positive results are obtained from this RCT, the PRISM-AC intervention can be recommended as an affordable, scalable skills-based psychosocial intervention for AYAs with advanced cancer. Moreover, the implementation of this trial can provide information to researchers about conducting psychosocial intervention trials with a pediatric advanced cancer population.

## Figures and Tables

**Table 1:** PRISM Modules

Topic	Details	Format
1. Managing Stress	Mindfulness techniques, relaxation strategies, obtaining social support	One-on-One
2. Goal-setting	Setting specific, realistic, desirable goals, planning for roadblocks	
3. Positive Reframing	Recognizing negative self-talk, replacing with positive, realistic, manageable ones	
4. Meaning Making	Identifying benefits, purpose, meaning, or legacy from cancer experience	
5. Voicing My Choices	Communication about values, preferences, and feelings about care	
6. Coming Together	Discussion about what was learned, what helped, what they can do to help	Family meeting
7. Boosters	In-person/digital/video conference modules to practice, further develop, and track skills.	One-on-One
8. Practice Opportunities	App-based modules to practice and further develop skills (also available in paper form)	Digital or Paper

Note: Sessions are delivered approximately every 1–2 weeks, arranged in advance in conjunction with hospital visits.

## Data Availability

The datasets generated and/or analyzed during the current study are not publicly available due to participant privacy concerns but are available from the corresponding author on reasonable request.
